# In Situ Surface Defect Detection in Polymer Tube Extrusion: AI-Based Real-Time Monitoring Approach

**DOI:** 10.3390/s24061791

**Published:** 2024-03-10

**Authors:** Chun Muk Jo, Woong Ki Jang, Young Ho Seo, Byeong Hee Kim

**Affiliations:** 1 Major in Mechatronics Engineering, Department of Mechanical Convergence Engineering, Kangwon National University, 1, Kangwondaehak-gil, Chuncheon-si 24341, Gangwon-do, Republic of Korea; 2 Department of Smart Health Science and Technology, Kangwon National University, 1, Kangwondaehak-gil, Chuncheon-si 24341, Gangwon-do, Republic of Korea

**Keywords:** smart manufacturing, polymer, real-time vision monitoring, object detection, defect detection, AI identification, YOLOv5, aging workforce

## Abstract

While striving to optimize overall efficiency, smart manufacturing systems face various problems presented by the aging workforce in modern society. The proportion of aging workers is rapidly increasing worldwide, and visual perception, which plays a key role in quality control, is significantly susceptible to the impact of aging. Thus it is necessary to understand these changes and implement state-of-the-art technologies as solutions. In this study, we conduct research to mitigate the negative effects of aging on visual recognition through the synergistic effects of real-time monitoring technology combining cameras and AI in polymer tube production. Cameras positioned strategically and with sophisticated AI within the manufacturing environment promote real-time defect detection and identification, enabling an immediate response. An immediate response to defects minimizes facility downtime and enhances the productivity of manufacturing industries. With excellent detection performance (approximately 99.24%) and speed (approximately 20 ms), simultaneous defects in a tube can be accurately detected in real time. Finally, real-time monitoring technology with adaptive features and superior performance can mitigate the negative impact of decreased visual perception in aging workers and is expected to improve quality consistency and quality management efficiency.

## 1. Introduction

In this era characterized by continuous technological advancements, industries are increasingly paying attention to implementing smart manufacturing systems. While striving to optimize overall efficiency, these systems confront various problems presented by the aging workforce in contemporary society. Changes in human physical and cognitive abilities are inevitable owing to aging, and it is necessary to fully understand these changes and implement state-of-the-art technologies as solutions [1,2,3]. Among these changing conditions, the foremost concern lies in the diminishing visual perception capabilities of aging workers in the manufacturing industry. Advances in healthcare and the increase in life expectancy have precipitated a visible surge in the proportion of older workers either joining the workforce or prolonging their tenure. In Europe, the percentage of the working-age population in the 15–24 age group is expected to drop by 5% by 2040 compared with 1990. In contrast, the 55–64 age group is expected to increase by nearly 6% [4]. It is predicted that by 2025, the proportion of working people over the age of 50 years will be 32% in Europe, 30% in North America, 21% in Asia, and 17% in Latin America. Consequently, many aging workers are affected by age-related problems [5]. This demographic change directly impacts productivity and efficiency because of the potential decline in the physical activity and cognitive ability of aging workers [6,7,8]. In this context, visual perception, which plays a key role in quality control, is susceptible to the effects of aging [5]. Alterations in vision and cognitive processing contribute to a reduction in workers’ ability to identify and monitor products and defects visually. Real-time camera monitoring can be implemented as a solution to this limitation [9,10]. Installing cameras at strategic locations within the manufacturing environment can monitor process errors in real time. In addition, videos can be analyzed in detail using sophisticated artificial intelligence (AI) algorithms to promote real-time defect detection and identification, enabling an immediate response from shopfloor workers. The combination of AI and real-time monitoring surpasses the simple identification of defects and explores solutions to improve the market competitiveness of manufacturing companies [11,12]. The convergence of these two technologies aims to facilitate basic research on simplifying and enhancing defect detection efficiency, overcoming limitations as the visual perception ability of aging workers decreases [1,13,14]. Furthermore, the adaptive nature of a system allows it to evolve and learn over time, perpetually refining its accuracy and performance. Moreover, implementing such an innovative system not only lessens the negative impact of aging on visual perception ability in manufacturing industries but also assists in improving the accuracy and efficiency of defect detection and quality control. Therefore, as the workforce navigates the inevitability of aging, the adaptability of manufacturing industries becomes more important. Hence, the proposed paradigm of real-time monitoring systems, seamlessly interweaving cameras, and AI has emerged as a solution and a transformative force set to address the decline in visual recognition skills among aging workers. Therefore, in this study, we researched visual recognition technology to mitigate the adverse effects of aging among workers through the synergistic effects of real-time monitoring technology that combines cameras and AI in polymer tube production.

Hence, we proposed solutions to address issues such as improving the working environment for aging workers, facilitating adaptation to the work environment in the manufacturing sector. The main objective of this study is to explore various methods for the perfect integration of aging workers and new technologies, aiming to address the visual perception decline in aging workers through technological means. Accordingly, we proposed a solution to the visual perception decline issue among aging workers through the convergence of a real-time monitoring system, cameras, and AI. The proposed solution was applied to the field of polymer tube manufacturing, where defects are detected only with the worker’s eye. A reliable quality assurance method based on advanced quality inspection technology is necessary to overcome the decline in the visual perception and monitoring abilities of aging workers in the manufacturing industry.

Research on detecting surface defects using AI and cameras is currently being carried out in diverse sectors, including metal and polymer compound manufacturing. In many studies, line scan cameras are positioned either solely at the top or at both the top and bottom of the inspection object, allowing the detection of defects with flat cross-sections [15,16]. Additionally, the direct lighting method often causes disturbances. Attempts have been made to alleviate these disturbances using monochrome cameras, yet this approach has its drawbacks, such as creating a detection screen that is challenging for workers to recognize or causing distortion during image processing.

In contrast, our study employs a distinct approach. Through careful camera arrangement and an indirect lighting environment, our methodology can be adapted for inspecting objects with circular shapes or those with non-uniform reflection characteristics. Furthermore, by incorporating advanced technology capable of detecting and intuitively displaying surface defects with high performance in the same RGB area as reality, we aim to overcome the challenges faced by manufacturing fields and aging workers.

The most significant contribution of this study is the development of a new understanding and solutions for addressing visual perception decline issues among aging workers. Our results aim to expand knowledge in the manufacturing industry and address the negative impact of aging on workers by leveraging the synergistic effect of real-time monitoring technology combining cameras and AI in polymer tube manufacturing.

## 2. Experimental Method

### 2.1. Defect Detection Target

**Figure 1:**
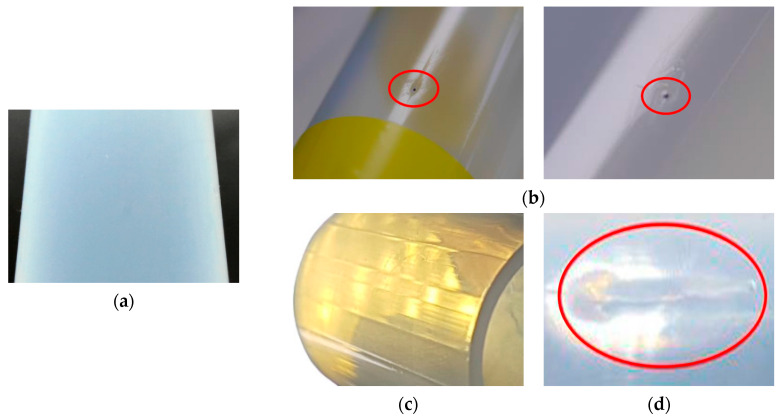
Tube surfaces: (**a**) normal; (**b**) inclusion; (**c**) scratch; (**d**) lumpy surface.

Among polymers, perfluoroalkoxy (PFA) tubes, known for their excellent chemical and corrosion resistances, are widely used to transport chemical substances [17]. However, typical defects occur on the tubing surface during the extrusion of PFA tubes. As shown in Figure 1b–d, the defects exhibit unique characteristics depending on their causes [18,19,20]. These defects can directly affect the manufacturing quality and durability of the PFA tubes [21]. Moreover, they can pose a fatal threat to worker safety in an industrial setting that handles hazardous substances and may lead to industrial accidents [22,23].

In this study, we aimed to identify and detect defects that frequently occur on the surface of PFA tubes using a real-time monitoring technology that combines cameras and AI. Figure 1a shows the surface of a flawless translucent normal tube. Figure 1b shows an inclusion formed during the tube extrusion process when foreign substances, such as dust, adhere to the hot surface of the tube and undergo carbonization. Figure 1c shows a scratch that occurs because of physical friction between the tube surface and another object. Compared to the normal tube surface, inclusions appear as black spots on the tube surface, whereas scratches are visible as white lines.

### 2.2. AI Algorithm for Defect Detection

Previous studies have aimed to identify and detect the quality or defects of products using cameras and image-processing algorithms for many years [24,25]. However, the filter-based algorithm used in image processing is a simple filtering algorithm that extracts the characteristics of the data by filtering based on a threshold, and its performance depends on the applied threshold. However, its detection performance is limited when the inspection target has uneven reflection characteristics or the distinction of defect areas is ambiguous. Furthermore, surface defects occurring at industrial sites can be small, and multiple defects may occur simultaneously. Therefore, relying solely on filtering and image processing for defect detection is inappropriate.

**Figure 2:**
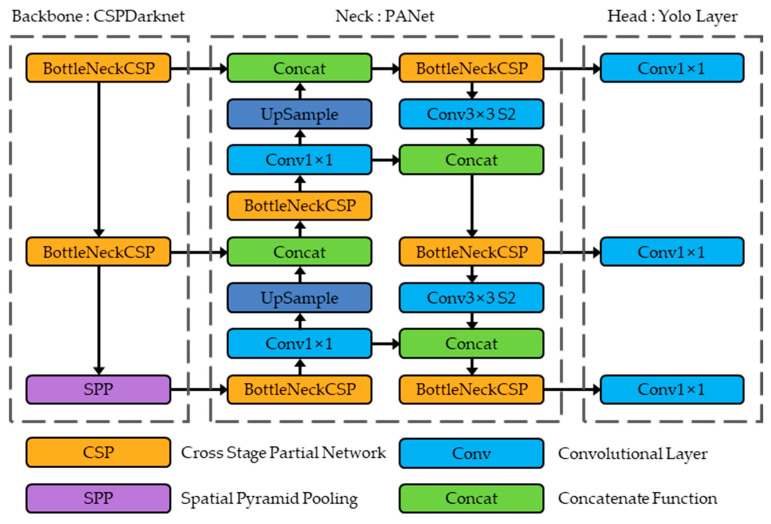
Architecture of YOLOv5 [26].

In this study, we implemented real-time monitoring technology using YOLOv5 (You Only Look Once version 5), an artificial neural network for object detection in a one-stage detector. Figure 2 depicts the architecture of YOLOv5 [26]. Its advantages include excellent detection performance and speed, better than the R-CNN series of algorithms that evolved as two-stage detectors in the early object detection field [26,27]. This AI algorithm is suitable for the real-time detection of simultaneous defects on the surfaces of PFA tubes, which are extruded at speeds ranging from a minimum of 0.004 to 0.012 m/s, by enabling the classification and localization of defects precisely and rapidly.

### 2.3. Development of Monitoring Housing

**Figure 3:**
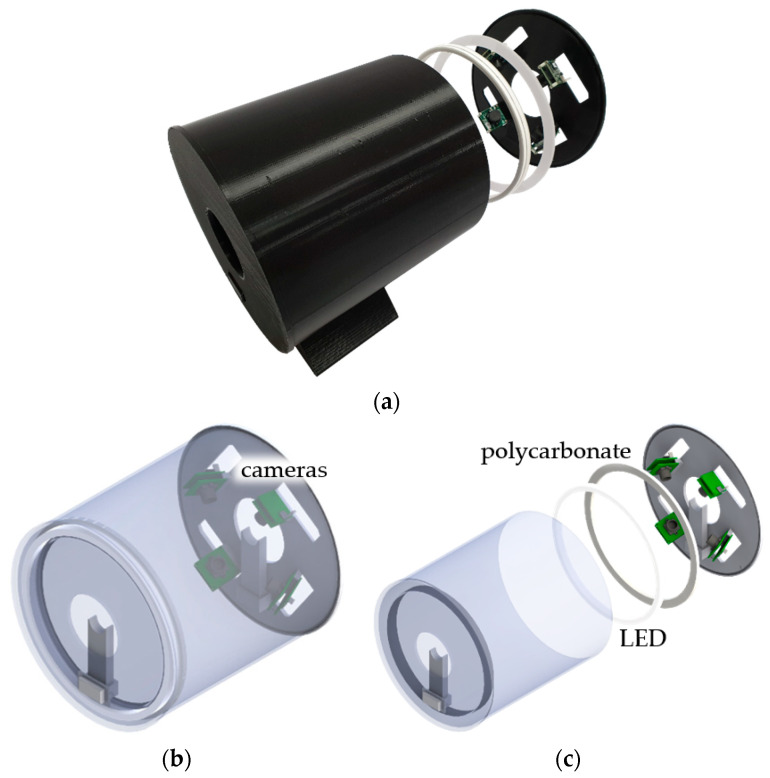
Monitoring housing: (**a**) prototype; (**b**) assembly diagram; (**c**) decomposition diagram.

Figure 3 depicts a cylindrical (Φ 210 × 206 mm) monitoring housing prototype produced using a 3D printer. It was designed to prevent and block disturbance factors that can obscure the characteristics of PFA tubes’ surface defects. Composite resins, metals, and other materials, including PFA tubes, exhibit uneven reflective properties owing to their surrounding environment and lighting conditions. Therefore, considering the reflective characteristics of the subject under inspection, a mining and monitoring environment for defect image data should be designed [28]. The interior of the monitoring housing was constructed using black PLA filaments to create a dark room environment, ensuring that it was not affected by external lighting. Furthermore, the ring polycarbonate (Φ 200 mm) was placed on the front of the surface-emitting LED (Φ 190 mm) with no prominent light spots, minimizing the impact of interior lighting by indirect lighting conditions and uniform light diffusion.

A USB camera module (See3CAM_CU135) was positioned to enclose the PFA tube to mine and monitor the defect image data. Optical and physical design elements, such as the minimum focus distance, field of view, and reflective properties, were simultaneously considered. Four cameras (*n* = 4) were equally spaced perpendicular to the direction of tube movement, allowing for 360° simultaneous monitoring of the tube circumference.

Furthermore, the monitoring housing was designed modularly, allowing for easy adjustment of the number of cameras (*n*). This is applicable to the surface defect monitoring of PFA tubes with various outer diameters ranging from approximately 3 to 60 mm.

**Figure 4:**
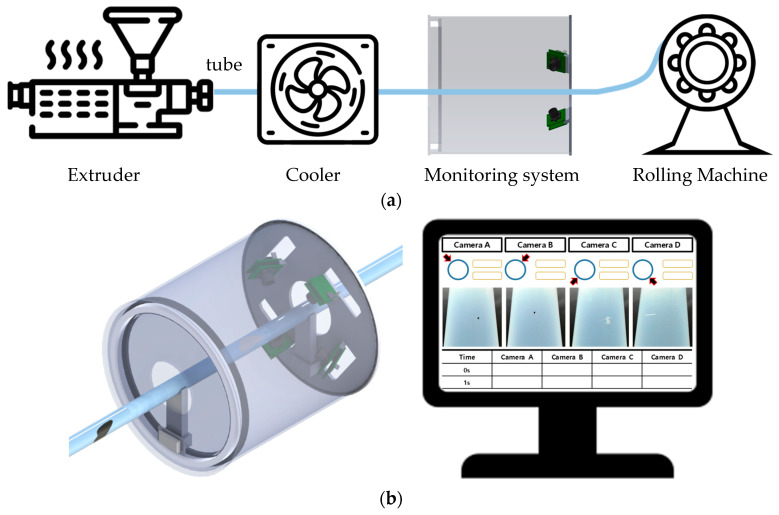
Schematic of a real-time monitoring system: (**a**) integrated into the production process; (**b**) defect detection notification screen.

The real-time monitoring system, as shown schematically in Figure 4a, can be integrated into the production process without hindering the operation machinery. The detection and notification of defects through real-time monitoring, as shown in Figure 4b, can help workers to respond promptly.

### 2.4. Defect Monitoring Technology Implementation

**Figure 5:**
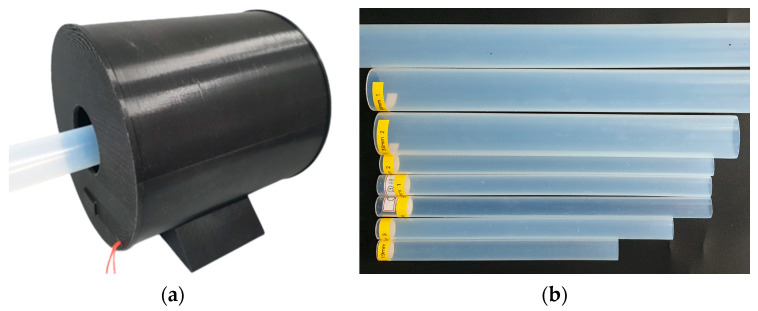
Data mining environment and samples: (**a**) data mining using monitoring housing; (**b**) surface defects samples.

**Figure 6:**
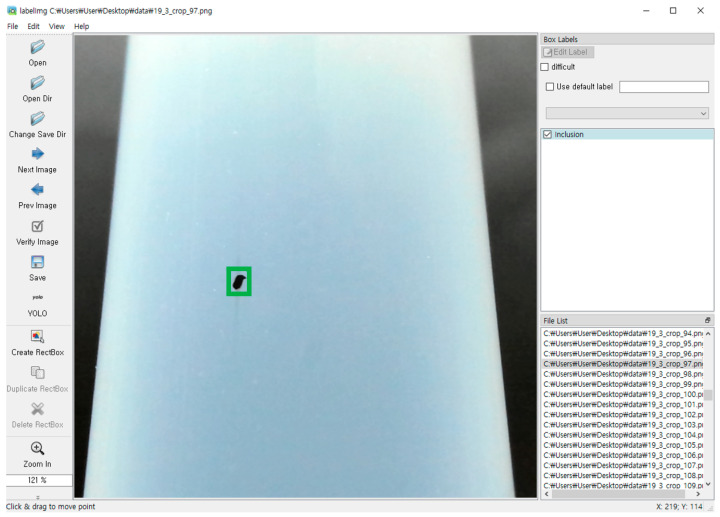
Defect labeling using LabelImg tool.

Figure 5a shows the environment used for mining surface defect data. In Figure 5b, samples of surface defects found on PFA tubes within an industrial setting are present. These defects were captured by a camera located within the monitoring housing, resulting in a dataset of 2000 defect images (1000 inclusions and 1000 scratches). As illustrated in Figure 6, the distinct features of these defects (inclusions and scratches) were meticulously specified with a ground truth bounding box using the LabelImg Tool for supervised learning. This process enabled the trained object detection algorithm to effectively generalize to various defect types and accurately predict new or untrained defects.

The performance of an artificial neural network trained on insufficient, biased, or monotonous data and lacking prominent features is not optimal. This common error in AI networks is called overfitting or underfitting [29]. Thus, defect image data should be mined and composed to prevent overfitting and underfitting.

**Figure 7:**
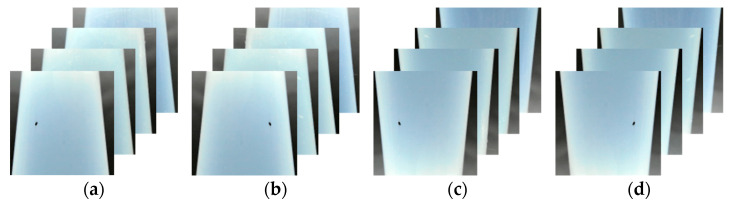
Data augmentation of inclusion: (**a**) original data; (**b**) left/right flip; (**c**) up/down flip; (**d**) 180° rotation.

**Figure 8:**
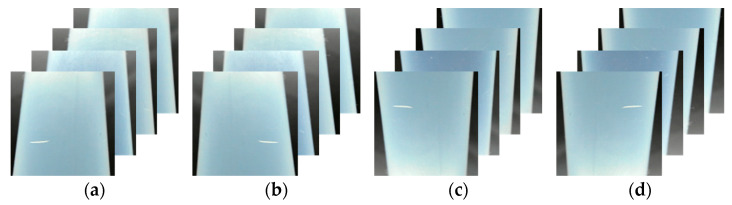
Data augmentation of scratch: (**a**) original data; (**b**) left/right flip; (**c**) up/down flip; (**d**) 180° rotation.

Several studies have demonstrated that data augmentation through image processing, such as rotation, flipping, blurring, brightness, and contrast adjustment, improves the performance of AI networks in common error prevention [30,31,32]. Rotation and flipping are geometric processing techniques which transform image data pixels using spatial and geometric calculations such as scaling, rotation, and translation. Figure 7 and Figure 8 show the augmented defect images created using the horizontal flip, vertical flip, and 180° rotation techniques. In this study, 8000 defect image data points were constructed using geometric processing for data augmentation. In addition, the training, validation, and test data were divided into a 6:2:2 ratio to ensure that the data, including the characteristics of defects, would be stratified and used for the supervised learning of the object detection AI networks. The training parameters were an epoch of 1000 and batch size of 32, and the specifications of the PC were Intel i9-10940X @ 3.30 GHz, 128 GB RAM, NVIDA GeForce RTX 2080Ti VRAM 11 GB.

### 2.5. Validation of Trained Model

The performance of the trained object detection model was verified and evaluated using K-fold cross-validation and mean average precision (mAP). K-fold cross-validation is a method that divides an entire dataset into k groups. It iteratively trains an AI network by designating each group as training, validation, or test data to ensure a reliable performance assessment [29,33]. The advantage of K-fold cross-validation is that it provides a more accurate estimate of a model’s performance than simply using a single train–test split. It also helps reduce the impact of random variation in the data by averaging the results over multiple iterations. In addition, K-fold cross-validation helps identify potential problems, such as overfitting or underfitting in the model. In this study, we validated the performance of the object detection model using five iterations. Stratified K-fold cross-validation was used to partition the groups. Stratified K-fold cross-validation ensured that the class proportions in each group were approximately the same as the class proportions of the entire dataset to ensure that the features of the surface defects were not split in a biased manner.

The detection performance of the object detection model was evaluated using mAP K times. The mAP is a validation metric that allows a quantitative comparison of the performance of AI networks using a confusion matrix [29]. The object detection algorithm was trained and validated using the training and validation data, and the mAP was measured using test data that were not used for training each time.

## 3. Experimental Results

Defect image data were mined in a uniform indirect lighting environment inside a monitoring housing prototype manufactured using a 3D printer and utilized for training. In an industrial setup, the uniform indirect lighting environment of the monitoring housing can be maintained robustly under uneven lighting conditions.

**Figure 9:**
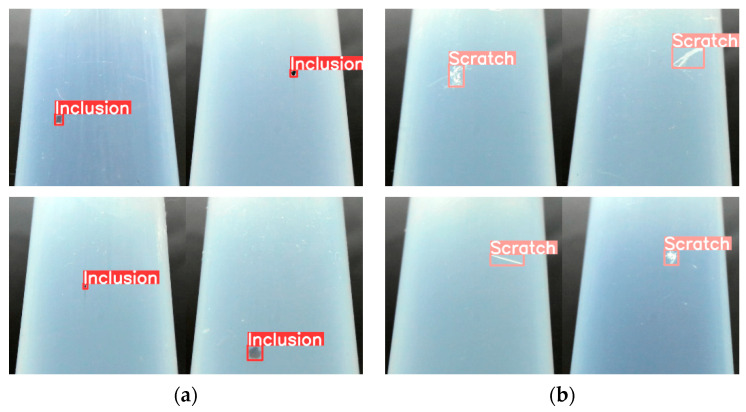
Results of defect detection: (**a**) inclusion; (**b**) scratch.

**Figure 10:**
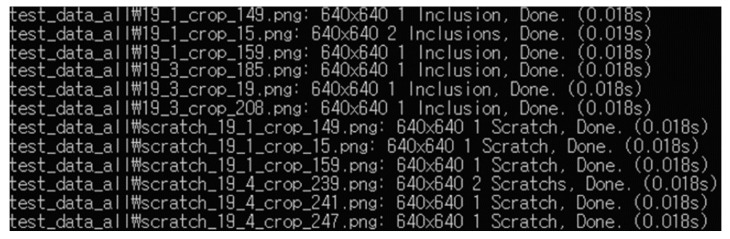
Identification time of monitoring system.

Figure 9 shows the results of the trained object detection AI network model for detecting defects in the test data. The model was verified to classify inclusions and scratches accurately and to indicate the location of the detected defects. Figure 10 shows that the identification time was approximately 20 ms, and it was possible to identify and assess the surface defects on the PFA tubes at approximately 30–50 frames per second. A multicamera system that allows simultaneous monitoring of the entire circumference of the tube was designed to ensure the uninterrupted operation of the tube production facility. This system was demonstrated to enable real-time monitoring when a tube is extruded.

Data augmentation through image processing and stratified K-fold cross-validation prevents common errors and improves the performance of AI networks, resulting in reliable outcomes. Table 1 shows the mAP@.5 measured at K = n (n = 1, 2, 3, 4, 5) by K-fold cross-validation. It demonstrated excellent performance with approximately 99.32% accuracy on the training data and approximately 99.24% accuracy on the test data. Furthermore, the vast amount of data accumulated from defect detection can be a decisive advantage of the AI-network-based real-time monitoring technology proposed in this study, allowing for a continuous improvement in defect detection performance.

As stated above, excellent detection performance and technical validity were evaluated and demonstrated by providing objective metrics and quantitative values through mAP measurements and real-time defect-detection tests.

## 4. Conclusions

In this study, we presented a camera- and AI-network-based real-time monitoring technology for impairments in defect identification and monitoring abilities to propose a solution to reduce the decline in visual perception abilities owing to the aging workforce. The feasibility of this approach was experimentally validated. The following conclusions were drawn from this study:

- With the convergence of appropriate camera placement and AI networks, a synergistic effect can be achieved to facilitate the prompt response of aging workers to defects.
- An immediate response to defects minimizes facility downtime and enhances the productivity of manufacturing industries.
- Real-time monitoring technology with adaptive features and superior performance can mitigate the negative impact of decreased visual perception in aging workers and is expected to improve quality consistency and quality management efficiency.
- By implementing sophisticated yet simple real-time monitoring technologies, manufacturing industries can overcome limitations and promote coexistence with aging workers, securing market competitiveness.

This technology can be implemented to detect inclusions, scratches, and other defects, such as porosity, that may occur during extrusion. Furthermore, in a follow-up study, we plan to compare the results using other RPN (Region Proposal Network) algorithms, such as Faster R-CNN, in addition to the YOLO algorithm. Finally, as suggested in this research, AI-network-based real-time monitoring technology is expected to be scalable for developing surface defect detection monitoring technology for polymers such as PFA tubes and shape-critical materials such as plastic products or semiconductor components.

## Figures and Tables

**Table 1:** mAP@.5 of the train and test data.

Iteration	mAP of Train Data (%)	mAP of Test Data (%)
K = 1	99.4	99.3
K = 2	99.4	99.4
K = 3	99.5	99.3
K = 4	99.4	99.2
K = 5	98.9	99
Average	99.32	99.24

## Data Availability

Data are contained within the article.
